# Meeting of the National Association for the Prevention of Tuberculosis

**Published:** 1905-05-27

**Authors:** 


### MEETING OF THE NATIONAL ASSOCIATION FOR THE PREVENTION OF TUBERCULOSIS.

Having for its object the discussion of a matter so intimately connected with the physical life and strength of the nation, the meeting at the Mansion House of the National Association for the Prevention of Tuberculosis, on the 17th inst., was naturally one of great interest. The chair was taken by the Lord Mayor. The meeting had been convened for the purpose of appealing to the public for funds to aid the association in their work, to explain what it has already done to check the disease, and what, with strengthened finances, it hopes to do in the future.

Sir William Broadbent, M.D., F.R.S., chairman of the council, gave some startling figures showing the ravages which consumption has made amongst the population. It is not, as he particularly remarked, the decrepit and unfit who are struck down, but the nation's strongest and best. The old idea of consumption, that it is a family inheritance, and therefore unavoidable, holds only a minimum of truth. The chief predisposing causes of consumption are deficient and bad food, exhaustion, overwork, vice, alcohol, and, above all, close and stuffy rooms, and windows close shut at night. The most potent factor in the spread of the disease is expectoration. Millions of tubercle bacilli are expelled with the patient's expectoration. Sir William described the particular danger of spitting in public-houses, where the expectoration, drying into dust, rises and spreads destruction broadcast. Milk, too, he declared, owing to the liability of cows to tuberculosis, is a terribly fruitful source of contagion to children, and it is a sad fact that amongst the little ones alone the mortality from consumption has not lessened during late years.

Notification to the health authorities when consumption is present in a household is a measure so desirable that Sir W. Broadbent hopes it will soon be made compulsory. The sanatoria for the cure of consumption do most excellent work, and the speaker ascribes the recent reaction against them to the unreasonableness of the public, who expect miracles to be performed on hopeless cases.

The Association are anxious that the Metropolitan Asylums Board should deal also with tuberculosis. They think that a central bureau should be established where all inquiries, both regarding consumption and the efforts of the Association to check it, could be obtained. Immediate financial aid, however, is indispensable for a continuance of the successful operations of the Association.

The Earl of Derby and Sir James Crichton Browne having addressed the meeting, the following resolution, which was unanimously carried, was proposed by Sir Homewood Crawford, seconded by Mr. Felix Schuster, and supported by Mr. G. Harewood, M.P., and Dr. Newman (medical officer of health for Finsbury):—

"That this meeting fully recognises the high importance to the community of the objects of the National Association for the Prevention of Consumption, and pledges its earnest support to the Association in this special appeal for £10,000, so urgently needed for the immediate prosecution and extension of its work."

Sir Herbert Maxwell, M.P., moved, and Sir James Blyth seconded, a vote of thanks for the presidency of the Lord Mayor.

